# Using topical imiquimod for the management of positive in situ margins after melanoma resection

**DOI:** 10.1002/cam4.402

**Published:** 2015-01-26

**Authors:** Amrita S Pandit, Erik J Geiger, Stephan Ariyan, Deepak Narayan, Jennifer Nam Choi

**Affiliations:** 1 Department of General Surgery, Western Connecticut Health NetworkDanbury, Connecticut, 06810; 2 Section of Plastic and Reconstructive Surgery, Yale University School of MedicineNew Haven, Connecticut, 06520; 3 Department of Dermatology, Yale University School of MedicineNew Haven, Connecticut, 06520

**Keywords:** Cutaneous, imiquimod, immunomodulator, melanoma, melanoma in situ

## Abstract

The treatment of melanoma in situ (MIS) is controversial with current standard of care being surgical excision with clear margins. Alternative topical therapy with imiquimod has been proposed in recent studies as a possible treatment for MIS. This study aimed to evaluate the use of topical 5% imiquimod as an alternative approach for the treatment of residual melanoma in situ after surgical resection of the primary lesion. A retrospective chart review of all patients treated with topical 5% imiquimod for residual MIS following standard resection with 5–10 mm margins at Yale-New Haven Hospital from 2008 through 2013 was performed. The pre- and posttreatment results were confirmed by diagnostic tissue biopsy. Twenty-two patients were included in the study. One of these 22 patients was lost to follow up. Twenty patients (95%) had complete resolution of their residual MIS and 1 patient did not respond to imiquimod (5%). No reports of recurrences were noted at the treatment sites. For patients with residual melanoma in situ after the initial excision, topical 5% imiquimod appears to be a reasonable alternative treatment with good clinical and histopathologic success rates.

## Introduction

Melanoma in situ (MIS) refers to early noninvasive melanoma confined to the epidermis and accounts for ∽27% of all melanomas 1. When left untreated, MIS may progress to invasive melanoma, with the lentigo maligna subgroup of MIS carrying up to a 4.7% lifetime risk of developing an invasive component 2. Therefore, MIS must be treated optimally to prevent its progression. The treatment options for MIS are controversial. The current standard of care based on consensus opinion is wide excision with 5–10 mm margins 3–5. However, experts note that particularly in cases of large MIS, lentigo maligna type, surgical margins >0.5 cm may be necessary to achieve histologically negative margins and careful histologic evaluation of surgical margins is strongly recommended 4,5. While various techniques have been used to achieve complete histologic margin control, such as permanent section total peripheral margin control and Mohs micrographic surgery, no prospective randomized data are available to make specific recommendations 4. The nonsurgical options for treatment of MIS include radiotherapy, cryotherapy, laser, and electrodessication and curettage. These nonsurgical options are generally reserved for elderly patients who are unsuitable for surgery, for patients with extensive involvement of the skin that is not amenable to surgical intervention given the likelihood of a poor cosmetic outcome, or when unresectable areas of the face are involved. Multiple pharmacological agents such as 5-fluorouracil, azelaic acid, retinoic acid derivatives, and interferon (IFN)-*α* have also been used for the treatment of MIS with success rates varying between 30% and 92% 6. There has been a growing number of reports suggesting that imiquimod, a topical immunomodulator, can be an effective alternative treatment for MIS 7–11.In fact, National Comprehensive Cancer Network's Clinical Practice Guidelines in Oncology for melanoma include the recommendation that imiquimod be considered as a treatment option for selected patients with positive MIS margins after optimal surgery 5. Imiquimod is commonly used for the treatment of actinic keratoses, superficial basal cell carcinomas, and genital human papilloma virus infections. Its mechanism of action is related to the induction of proinflammatory cytokines leading to a TH1 immune response 12. Imiquimod inhibits angiogenesis and rapidly increases peritumoral infiltration of natural killer (NK) cells and cytotoxic T-cells resulting in apoptosis of melanoma cells. Imiquimod activates macrophages and other cells via binding to cell surface receptors, such as toll-like receptor 7, and induces secretion of proinflammatory cytokines, predominantly interferon alpha (IFN-*α*), tumor necrosis factor alpha (TNF-*α*), and interleukin-12 (IL-12) 12–14.

We performed a retrospective analysis of 21 patients who underwent treatment with topical imiquimod for residual MIS after initial wide local resection of primary melanoma or MIS lesions. We hypothesized that topical imiquimod therapy alone would effectively treat MIS in this setting and clear histologic margins.

## Methods

We performed a retrospective chart review of patients treated with topical 5% imiquimod cream for MIS that had persisted after initial surgical excision of melanoma or melanoma in situ. All patients included in the review had undergone diagnostic biopsy at their initial presentation, followed by standard excision with 1-cm margins for Breslow depth ≤1 mm, 2-cm margins for Breslow depth of 2 mm and above, and a minimum of 5-mm margins for MIS. These patients were then found to have varying degrees of residual MIS at the excision margins through histologic analysis of the surgical specimens. Patients with residual MIS were offered topical application of 5% imiquimod cream over the involved area of skin or reresection. Patients who preferred imiquimod application over reresection were evaluated for application of imiquimod. The study was approved by the Yale University Human Investigation Committee. Informed consent was obtained from all the patients undergoing treatment after a discussion of the risks and benefits of treatment with imiquimod.

**Figure 1:**
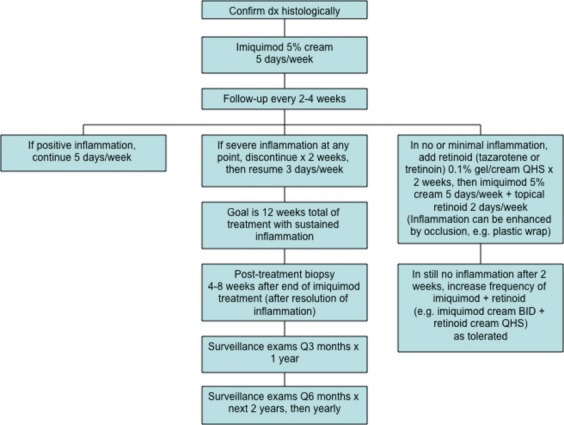
Protocol used at our institution to guide treatment of residual melanoma in situ margins after surgical resection.

Patients began imiquimod therapy with topical applications, typically starting at a frequency of 5 days each week. This regimen was gradually tailored based on each patient's clinical response. Figure1 outlines the protocol that guided each patient's treatment regimen. During the treatment period, the patients were monitored closely with regular follow-up, typically every 2–4 weeks. Pretreatment and posttreatment histological assessments were performed on all patients. The posttreatment biopsy was performed at ∽4–8 weeks following the last application of topical imiquimod cream, after treatment-induced inflammation was resolved. The posttreatment biopsy sites were chosen based on the location of residual MIS that was noted at the surgical excision margins. For example, if residual MIS was noted from 2 to 4 o'clock on a surgical margin, the biopsies were done at that site. Biopsies consisted of 4-mm punch biopsies, ranging in number from 2 to 4 at a time, depending on the size of residual MIS margin involvement. Immunohistochemical evaluation, such as MITF staining, was performed in any case which exhibited persistent melanocytes.

## Results

**Figure 2:**
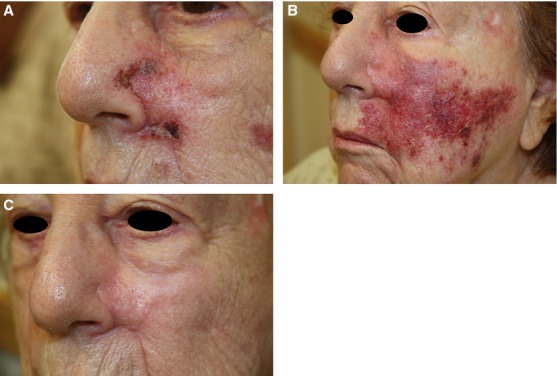
(A) 4 weeks into treatment with imiquimod 5% cream, (B) 11 weeks into treatment just before discontinuation of imiquimod cream, (C) 5 weeks after surveillance biopsies were performed, which revealed clearance. Patient continues to be in remission with no signs of local or metastatic recurrence 4.5 years after diagnosis.

A total of 22 patients having residual MIS at surgical margins after excision procedures for primary melanoma or MIS were treated with topical 5% imiquimod cream. Patient demographics and details of the primary lesions are provided in Table1. Twenty patients (95%) had an excellent response with complete resolution of their residual MIS at a mean follow-up period of 24 months. This was confirmed by a minimum of two 4-mm punch biopsies of the affected margins following the treatment. Table2 indicates the frequency and duration of topical imiquimod treatment, as well as the cutaneous inflammatory response of each patient during treatment and their associated clinical outcome. One patient (5%) was lost to follow-up. Another patient (5%) had persistent MIS after treatment with imiquimod. She was subsequently treated by reresection but was again found to have residual MIS at the margins. She is currently being monitored clinically with no evidence of repigmentation of the affected area. Figure2A–C shows representative photos of reactive inflammation during the course of imiquimod treatment for patient 3.

**Table 1:** Patient demographics and primary lesion details (all patients initially treated with surgical excision)

Patient number	Age	Sex	Site of lesion	Type of lesion (Breslow depth)^1^, ^2^
1	52	F	R frontal hairline	MIS
2	65	F	R cheek	Melanoma (0.1 mm)
3	92	F	L cheek	Melanoma (0.5 mm)
4	62	F	R lateral eyelid, R nose	MIS
5	49	M	R cheek	MIS
6	57	M	R eyelid	Melanoma (1.35 mm)
7	69	M	R parietal scalp	0.5 mm
8	80	F	L paramedian forehead	MIS
9	79	M	Dome of nose	Melanoma (0.9 mm)
10	75	M	L great toe	MIS
11	82	F	L ear	MIS
12	63	M	R upper cheek	MIS
13	84	F	L heel	Melanoma (0.55 mm)
14	87	M	L upper arm	Melanoma (0.75 mm)
15	75	M	Anterior scalp	Melanoma (2.5 mm)
16	75	M	Dorsum of nose	MIS
17	59	M	R neck	MIS
18	62	F	R shoulder	Melanoma (0.35 mm)
19	73	F	L cheek/temple	Melanoma (0.55 mm)
20	83	F	R index finger	MIS
21	82	F	L upper lip	MIS
22	56	F	R nasal ala	MIS

MIS, melanoma in situ; M, male; F, female; R, right; L, left.

1 Breslow depth indicated for invasive melanoma lesions only.

2 All patients initially treated with surgical excision.

**Table 2:** Imiquimod application regimen, treatment response, and clinical outcome for each patient

Patient number	Imiquimod frequency (days/week)^1^	Duration (weeks)^1^	Inflammatory response	Disease outcome	Surveillance biopsy results	Follow-up duration (months)
1	5	17	Severe	CCC	No MIS	52
2	5	4	Severe	CCC	Lost to follow-up	43
3	3–5	11	Severe	CCC	No MIS	47
4	5	6	Severe	CCC	No MIS	46
5	2–5	12	Severe	CCC	No MIS	46
6	3	40	Moderate	CCC	No MIS	29
7	3–5	12	Moderate—severe	CCC	No MIS	52
8	2–5	22^2^	Severe	PCC	No MIS	23
9	3–5	12	Severe	CCC	No MIS	11
10	3–5	12	Moderate	CCC	No MIS	6
11	3	24^2^	Moderate	CCC	No MIS	8
12	0.5–5	13	Moderate	CCC	No MIS	53
13	3	12	Severe	CCC	No MIS	9
14	3	12	Moderate	CCC	No MIS	18
15	5	12	Moderate	CCC	No MIS	7
16	3	10	Severe	CCC	No MIS	12
17	3	12	Severe	CCC	No MIS	6
18	3	6	Severe	CCC	No MIS	42
19	3–5	12	Severe	CCC	No MIS	24
20	5^3^	7	None	Failure	Persistent MIS	10
21	3–7	12	Severe	CCC	No MIS	15
22	3–5	16	Severe	CCC	No MIS	7

CCC, complete clinical response; PCC, partial clinical response; MIS, melanoma in situ.

1 Imiquimod dosing regimen was titrated to balance inflammation against patient tolerance.

2 Residual MIS after one round of treatment, so patient underwent an additional 12 week course.

3 No inflammation achieved even with BID dosing.

## Discussion

Our study evaluated the use of topical imiquimod cream in the management of MIS existent after the surgical resection of primary melanoma or MIS lesions. We hypothesized that a treatment regimen of 12 weeks—tailored based on individual patient's tolerance to the inflammatory response created by the therapy—would be sufficient to clinically and histologically clear the surgical margins. We were able to achieve a 95% complete clinical response rate in our cohort of 22 patients. Although the current recommended management of MIS is wide local excision with a minimum of 5–10 mm margins 4, larger lesions of the head and neck, commonly of the lentigo maligna type, frequently require larger surgical margins to achieve histologic clearance. Our data provide further support for the use of topical imiquimod as a nonsurgical alternative in the treatment of MIS in patients who are either not suitable candidates for reresection or who wish to avoid further surgery for other reasons.

The degree of inflammation around the site of disease may be used as a reliable predictor of outcome 9,15. In one series of patients, there was a statistically significant association between visible association and histopathologic clearance 15. In our series of 22 patients, one patient failed to show any inflammatory reaction to imiquimod. It was this same patient who was the only subject to have persistent positive margins with MIS despite the treatment for 12 weeks. Our data support the observation from previous reports that a positive inflammatory reaction signals a better clinical outcome in these patients.

The clinical treatment protocol for topical imiquimod cream is not standardized, and the ideal frequency and duration of therapy remains an area of active investigation. Some reports have described once or twice daily applications for durations varying between 2 and 88 weeks 11,16–18, depending on the individual response. Other reports have suggested that 5 days of topical application of 5% imiquimod cream for 12 weeks is effective 6,9,15. Most of our patients were treated 3–5 times per week for a minimum of 12 weeks. However, in our clinical experience, we found that the treatment regimen has to be individualized with close follow-up due to the varying degrees of inflammation and pain tolerance in each patient.

The common side effects of imiquimod application include local reactions such as burning, pain, tenderness, vesicular eruptions, and ulcerations. These reactions usually subside after discontinuation of therapy. Pigmentation changes including vitiligo or hyperpigmentation have also been reported. Other rare side effects such as cytokine release syndrome can occur. This syndrome manifests with constitutional symptoms including headache, gastrointestinal upset, fever, and malaise 19,20. Chronic neuropathic pain, autoimmune spondyloarthropathy, renal failure, and serious conditions such as erythema multiforme, Stevens Johnson syndrome, and cutaneous lupus erythematosus have also been reported with topical imiquimod therapy 19,20. One patient in our trial developed severe acute depression secondary to the use of imiquimod requiring discontinuation of treatment, which led to the resolution of her depressive symptoms.

Some reports also have recommended the use of keratolytic agents such as tazarotene as an adjunct to imiquimod 21. Treatment with keratolytic agents can be initiated 2 weeks prior to initiation of imiquimod and/or used concurrently with imiquimod. The use of tazarotene is thought to increase the efficacy of imiquimod cream by enhancing drug penetration through the disruption of the stratum corneal barrier 21. Previous reports have suggested the optimal time for the posttreatment biopsy is between 4 and 8 weeks after the last application of imiquimod (in order to give the local inflammatory response time to dissipate). The biopsies for our patients were typically taken during this interval, except for one patient who received his biopsy 1 week following completion of treatment. Interestingly, the biopsy results of this patient showed superficial and deep perivascular and periadnexal lymphoplasmacytic infiltrate with interface changes similar to that of discoid lupus erythematosus. These changes were attributed to the use of imiquimod, and have previously been reported 22. There was no histological evidence of residual MIS on this patient's biopsy.

There are limitations to this study inherent to all retrospective analyses. One limitation of our trial is interobserver variation in microscopic/histological diagnosis of MIS, although all biopsies were performed and interpreted at the same institution to minimize such variability. A second limitation of this study is the relatively short-term follow-up period, although we believe having a mean follow-up of 2 years still allows us to report meaningful clinical conclusions. All patients in our series continue to be under vigilant watch to monitor for any signs of recurrence. An additional limitation is that the post-treatment sampling consisted of two to four 4-mm punch biopsies to assess for disease resolution at a histologic level, which is not as accurate as reexcision of the entire surgical margin with complete histologic assessment of the tissue specimen. Punch biopsies lend to possible sampling error and may miss foci of residual MIS. Regular and long-term follow-up, with a low threshold for further diagnostic biopsies, is strongly recommended for these patients.

## Conclusion

Our data support the use of topical imiquimod cream as a safe, effective alternative to surgical reexcision in selected individuals with positive MIS margins after initial surgical resection of melanoma or MIS. Although our series shows promising results, larger studies need to be performed with longer follow-up periods to confirm the utility of imiquimod in such cases.

## Conflict of Interest

None declared.
